# Kidney stone formers have more renal parenchymal crystals than non-stone formers, particularly in the papilla region

**DOI:** 10.1186/s12894-018-0331-x

**Published:** 2018-03-12

**Authors:** Atsushi Okada, Shuzo Hamamoto, Kazumi Taguchi, Rei Unno, Teruaki Sugino, Ryosuke Ando, Kentaro Mizuno, Keiichi Tozawa, Kenjiro Kohri, Takahiro Yasui

**Affiliations:** Department of Nephro-urology, Nagoya City University Graduate School of Medical Sciences, 1 Kawasumi, Mizuho-cho, Mizuho-ku, Nagoya, Aichi 467-8601 Japan

**Keywords:** Kidney stones, Macrophages, Osteopontin, Oxidative stress, Tamm-Horsfall protein

## Abstract

**Background:**

We investigated the renoprotective ability of healthy people against kidney stone formation. To clarify intratubular crystal kinetics and processing in human kidneys, we performed a quantitative and morphological observation of nephrectomized renal parenchyma tissues.

**Methods:**

Clinical data and pathological samples from 60 patients who underwent radical nephrectomy for renal cancer were collected from June 2004 to June 2010. The patients were retrospectively classified as stone formers (SFs; *n* = 30, kidney stones detected by preoperative computed tomography) and non-stone formers (NSFs; *n* = 30, no kidney stone history). The morphology of parenchymal intratubular crystals and kidney stone-related gene and protein expression levels were examined in noncancerous renal sections from both groups.

**Results:**

SFs had a higher smoking rate (*P* = 0.0097); lower red blood cell, hemoglobin, and hematocrit values; and higher urinary red blood cell, white blood cell, and bacterial counts than NSFs. Scanning electron microscopy revealed calcium-containing crystal deposits and crystal attachment to the renal tubular lumen in both groups. Both groups demonstrated crystal transmigration from the tubular lumen to the interstitium. The crystal diffusion analysis indicated a significantly higher crystal existing ratio in the medulla and papilla of SFs and a significantly higher number of papillary crystal deposits in SFs than NSFs. The expression analysis indicated relatively high osteopontin and CD68, low superoxide dismutase, and significantly lower Tamm–Horsfall protein expression levels in SFs. Multivariate logistic regression analysis involving the above factors found the presence of renal papillary crystals as a significant independent factor related to SFs (odds ratio 5.55, 95% confidence interval 1.08–37.18, *P* = 0.0395).

**Conclusions:**

Regardless of stone formation, intratubular crystals in the renal parenchyma seem to transmigrate to the interstitium. SFs may have reduced ability to eliminate renal parenchymal crystals, particularly those in the papilla region, than NSFs with associated gene expression profiles.

## Background

Since the introduction of extracorporeal shock-wave lithotripsy in 1980, [1] fewer opportunities for open surgery have led to fewer chances for pathological investigations of kidney stone formation (KSF) using human kidney parenchymal tissue. Therefore, studies of human KSF tend to focus on urinary inorganic concentrations [2, 3] and epidemiological data, such as those investigating the relationship with diabetes [4] and metabolic syndrome [5]. Recent progress in endoscopic technology has directed attention to Randall’s plaque [6]; interstitial apatite crystal deposits beginning at the basement membranes of the thin loops of Henle seem to be sites of calcium oxalate (CaOx) stone formation [7–9]. However, intraparenchymal events involving the kinetics of intratubular crystals have not been elucidated.

Previous basic studies using hyperoxaluric-animal and cell-culture models led to the detection of morphological and genetic events in the renal parenchyma via the detection of stone matrix protein, [10] completion of the human genome project, and technological progress related to recombinant gene analysis [11–13]. In particular, the factors currently considered to affect calcium kidney stone formation are stone matrix proteins, cell injury caused by oxidative stress, monocyte/macrophage induction, and urinary stone inhibitors.

Osteopontin (OPN), the main component of stone matrix protein, is a glycoprotein present in human calcium-containing kidney stones [10] that may play an important role in crystal conversion to stones. OPN antisense-expressing cultured renal tubular cells demonstrate reduced aggregation of CaOx crystals and crystal-cell interactions [14] and OPN-knockout mice show reduced growth of renal crystals [12]. OPN is also involved in the formation of the organic layer of apatite plaque particles in the renal inner medulla of CaOx stone formers (SFs) [15]. Furthermore, renal tubular-cell injury caused by oxidative stress is essential for kidney stone formation [16]. Some studies have indicated that tubular-cell apoptosis caused by deviated free radicals and diminished superoxide dismutase (SOD) expression, [17] and collapsed organelles, including mitochondria and fragmented microvilli in the renal tubular lumen, lead to stone nidus formation [18]. We reported renal intratubular crystal elimination in a mouse model, increased expression of macrophage-related inflammatory genes in a DNA microarray analysis of stone-forming kidneys, [13] and phagocytosis of interstitial crystals by macrophages under transmission electron microscopy, [19] suggesting the kidney stone-preventive ability of macrophages by crystal processing. Moreover, Umekawa et al. [20] demonstrated that exposure to CaOx crystals promotes the expression of monocyte chemotactic protein-1 (MCP-1) and induces macrophage migration. Finally, Tamm–Horsfall protein (THP), a urinary inhibitor of stone formation, has been studied because THP-deficient mice demonstrate spontaneous calcium crystal formation [21].

The above experimental findings suggest that intratubular crystal formation involves several steps and that animal models may have the ability to eliminate the crystals. However, among the possible mechanisms of stone formation, these processes are thought to model Randall’s plug due to hyperoxaluria or cystinuria rather than Randall’s plaque. However, there is no definitive evidence to confirm this assumption. With the above in mind, we aimed to elucidate the intratubular crystal kinetics and processing in human kidneys using nephrectomized parenchymal tissues.

## Methods

### Patients

We obtained clinical data and pathological samples from 60 patients who underwent radical nephrectomy for stage I renal cell carcinoma (RCC) from June 2004 to June 2010. The Institutional Review Board of Nagoya City University Hospital approved the study design (Approval No. 551). The patients were retrospectively classified as SFs (30 patients with renal stones detected by preoperative computed tomography [CT]) and non-stone formers (NSFs; 30 age [± 1 year] - and sex-adjusted patients without renal stone and kidney disease history). In SFs, all stones were also detectable by abdominal X-ray and were presumed to be non-uric acid stones.

### Clinical data analysis

We evaluated basic clinical and pathological data, comorbidities, and lifestyle factors. The preoperative laboratory data analyses included complete blood count, coagulability tests, and biochemical analyses. Using spot urine sampling, qualitative analysis of specific gravity, pH, protein, and glucose and flow cytometry-based quantitative analysis of urinary red blood cells (RBCs), white blood cells (WBCs), epithelial cells, and bacteria were conducted.

### Aortic calcification index

Because of the similarity between atherosclerosis formation and kidney stone formation, [22] we calculated the aortic calcification index of both groups as the degree of calcification at the aortic arterial wall as follows: grade 0, none; grade 1, < 120 degrees of calcification; grade 2, ≥ 120 degrees but < 240 degrees of calcification; and grade 3, ≥ 240 degrees of calcification.

### Detection and quantification of renal crystal deposits

Paraffin-embedded tissue blocks prepared from formalin-fixed excised kidneys were sliced to a 4-μm thickness and stained with hematoxylin and eosin (H&E). Crystal deposits in the normal renal parenchyma were detected by polarized light optical microphotography of the H&E-stained samples. The number of crystal deposits was quantified by counting the crystals per 100 visual fields (magnification, × 100) in noncancerous sections of the renal cortex, medulla, and papilla and as the existing ratio (number of kidneys with crystal deposits/whole kidneys). CaOx crystals were detected by Pizzolato staining [23].

### Scanning electron microscopy (SEM) analysis

Dewaxed paraffin-embedded sections (4-μm thickness) were washed with a phosphoric acid buffer, re-fixed with 2.5% glutaraldehyde and subsequently with 2% osmium liquid, dehydrated in a 50–100% ethanol series, and embedded in epoxy resin. After sputtering a platinum filter on a stage, SEM specimens were prepared using electrical conduction. The crystal ultrastructure was then examined by SEM. The elemental spectra of the crystal deposits were determined by energy-dispersive X-ray spectroscopy (EDX).

### Immunohistochemistry (IHC)

IHC for OPN, SOD, CD68 (a macrophage surface marker), and THP was performed using 4-μm-thick cross-sections. The tissues were autoclaved for antigen activation at 121 °C for 5 min, blocked with 0.5% hydrogen peroxide in methanol for 30 min, washed with 0.01 M phosphate-buffered saline (PBS), and treated with skimmed milk in PBS for 1 h at room temperature. They were then incubated overnight at 4 °C with the following polyclonal antibodies: rabbit anti-human OPN (Immuno-Biological Laboratories Co., Ltd., Gunma, Japan), rabbit anti-human CD68 (Santa Cruz Biotechnology, Santa Cruz, CA, USA), rabbit anti-human THP (Santa Cruz Biotechnology), and goat anti-human SOD (Santa Cruz Biotechnology). The reacted antibodies were detected using a Histofine simple stain kit for goat or rabbit immunoglobulin G (Nichirei Biosciences, Inc., Tokyo, Japan) according to the manufacturer’s instructions.

### Quantitative reverse transcription-polymerase chain reaction (qRT-PCR) analysis

Total RNA from noncancerous kidney sections was extracted using NucleoSpin FFPE RNA (Macherey-Nagel GmbH & Co., Düren, Germany) according to the manufacturer’s instructions. All RNA samples were reverse-transcribed to complementary DNA with a High Capacity cDNA reverse transcription kit (Applied Biosystems, Life Technologies, Carlsbad, CA, USA). According to the annotation information of each gene, the TaqMan gene expression assay product, a 20× assay mix of forward and reverse primer sets, and TaqMan MGB probe (FAM dye labeled) with complementary sequences to each messenger RNA sequence were obtained. The qPCR was performed with the TaqMan Universal PCR master mix (404,437, Applied Biosystems) using the 7500 FAST real-time PCR system (Applied Biosystems). After denaturation at 95 °C for 10 min, PCR was initiated at 95 °C for 15 s and completed at 60 °C for 1 min. The reaction was repeated 45 times. The expression of each sample was determined as a ratio to the expression of the glyceraldehyde 3-phosphate dehydrogenase gene (*GAPDH*; internal control). TaqMan gene expression assay probe kits were used for the secreted phosphoprotein-1 gene (*SPP1*, encoding OPN; Hs00959010_m1), *SOD1* (Hs00533490_m1), *CD68* (Hs00154355_m1), uromodulin gene (*UMOD*, encoding THP; Hs00358451_m1), and *GAPDH* (Hs03929097_g1).

### Statistical analysis

We used the chi-square test using a 2 × 2 table to compare the comorbidities and lifestyle factors. The clinical and basic laboratory data were compared using Student’s *t*-test. Furthermore, the pathological data, aortic calcification grades, urinalysis results, number of crystal deposits, and messenger RNA expression levels were analyzed using the Mann–Whitney *U*-test. The categorical pathological patient data, comorbidity data, and lifestyle factor data were assessed using the chi-square test. Repeated-measures analysis of variance (ANOVA) was used to compare the renal crystal distribution between the groups. Based on each analysis, the extracted factors were evaluated for their relationship with kidney stone formation using multivariate logistic regression analysis. In these analyses, a *P*-value < 0.05 was considered to indicate a statistically significant difference.

## Results

### Clinical findings

The groups were not significantly different in terms of clinical data. No significant sex difference was detected between the groups (Table 1). Both groups were predominantly composed of patients with clear-cell RCC. There were no significant differences in pathological diagnoses, grades, stages, and affected sides between the groups (Table 2). Comorbidities did not differ between the groups (Table 3). However, SFs had a significantly higher smoking rate than NSFs (*P* = 0.0097).

**Table 1:** Clinical patient data

Parameter	Total, mean (SD)		*P*-value*	Men, mean (SD)		*P*-value*	Women, mean (SD)		*P*-value*
	SFs	NSFs		SFs	NSFs		SFs	NSFs	
Age (years)	62.1 (10.6)	61.7 (10.9)	0.8856	62.7 (10.3)	63.0 (10.3)	0.9336	56.6 (13.3)	57.6 (12.2)	0.8856
Height (cm)	164.6 (7.4)	165.3 (6.7)	0.7016	166.1 (6.6)	166.7 (5.9)	0.7282	156.8 (6.6)	157.6 (5.2)	0.8366
Weight (kg)	62.2 (12.1)	66.0 (10.5)	0.1933	64.0 (11.7)	67.2 (9.6)	0.2902	53.2 (10.4)	59.8 (13.8)	0.4181
Body mass index (kg/m^2^)	22.9 (3.4)	24.0 (3.7)	0.2149	23.2 (3.4)	24.0 (3.0)	0.3587	21.6 (2.9)	24.2 (6.6)	0.4455
Abdominal circumference (cm)	77.7 (8.1)	80.4 (8.3)	0.2027	78.2 (8.1)	80.5 (8.1)	0.3260	74.9 (8.0)	79.8 (10.2)	0.4243

*SD* standard deviation, *SFs* stone formers, *NSFs* non-stone formers

*P < 0.05 indicates statistically significant differences by Student’s *t*-test

**Table 2:** Pathological patient data

Parameter	Number of SFs (%)	Number of NSFs (%)	*P*-value*
Diagnosis			0.7879
Clear cell RCC	27 (90.0)	28 (93.3)	
Papillary RCC	1 (3.3)	1 (3.3)	
Chromophobe RCC	1 (3.3)	0 (0)	
Collecting duct carcinoma	1 (3.3)	1 (3.3)	
Grade			0.3529
1	8 (26.7)	9 (30.0)	
2	20 (66.7)	21 (70.0)	
3	2 (6.7)	0 (0)	
INF			0.5194
a	18 (64.3)	20 (69.0)	
b	10 (35.7)	8 (27.6)	
c	0	1 (3.4)	
pT			0.7249
1a	12 (40.0)	13 (43.3)	
1b	9 (30.0)	11 (36.7)	
2	2 (6.7)	1 (3.3)	
3a	2 (6.7)	3 (10.0)	
3b	5 (16.7)	2 (6.7)	
Side			0.0705
Right	12 (40.0)	19 (63.3)	
Left	18 (60.0)	11 (36.7)	

*SFs* stone formers, *NSFs* non-stone formers, *RCC* renal cell carcinoma

*P < 0.05 indicates statistically significant differences by the chi-square test

**Table 3:** Comparison of the comorbidities and lifestyle factors

Parameter	Number of SFs (%)	Number of NSFs (%)	*P*-value*
Comorbidity			
Hypertension	15 (50.0)	14 (46.7)	0.7961
Heart disease	4 (15.4)	3 (11.1)	0.6876
Cerebrovascular disease	0 (0.0)	2 (6.7)	0.1503
Diabetes	2 (6.7)	7 (23.3)	0.0706
Habituation			
Smoking	19 (63.3)	9 (30.0)	**0.0097**
Drinking	11 (36.7)	6 (20.0)	0.1520

*SFs* stone formers, *NSFs* non-stone formers

The bold number indicates a statistically significant difference (*P < 0.05) by the chi-square test for a 2 × 2 table

Although the RBC, hemoglobin, and hematocrit (Ht) values of SFs were within the normal limits, they were significantly lower than those of NSFs (*P* = 0.0290, 0.0360, and 0.0268, respectively; Table 4). The coagulation-related and blood biochemical data were not significantly different between the groups. Furthermore, no significant differences in urinalysis results were noted. However, SFs had significantly higher urinary RBC, WBC, and bacterial count values (*P* = 0.0343, 0.0117, and 0.0014, respectively); male patients had similar values for the above parameters (*P* = 0.0108, 0.0036, and 0.0010, respectively). Qualitative analysis of urinary protein and glucose levels did not yield significant differences between the groups (Table 5).

**Table 4:** Preoperative laboratory patient data

Parameter	Total, mean (SD)		*P*-value*	Men, mean (SD)		*P*-value*	Women, mean (SD)		*P*-value*
	SFs	NSFs		SFs	NSFs		SFs	NSFs	
WBC (× 10^3^/μL)	5.7 (1.4)	6.4 (1.8)	0.1172	5.9 (1.4)	6.7 (1.8)	0.0870	5.1 (0.9)	5.0 (1.2)	0.8366
Neutrophil (%)	62.1 (8.6)	62.4 (6.5)	0.8800	62.4 (8.9)	62.8 (6.0)	0.8742	59.3 (7.4)	61.1 (9.3)	0.8125
Eosinophil (%)	3.3 (3.3)	3.0 (2.3)	0.7481	3.6 (3.4)	3.1 (2.4)	0.5742	1.4 (0.80)	2.7 (1.9)	0.2588
Basophil (%)	0.4 (0.3)	0.5 (0.4)	0.3021	0.5 (0.3)	0.5 (0.4)	0.5532	0.2 (0.20)	0.5 (0.1)	0.0656
Monocyte (%)	5.2 (1.6)	5.5 (1.7)	0.5208	5.4 (1.4)	5.6 (1.2)	0.7515	3.6 (1.90)	4.9 (3.3)	0.5034
Lymphocyte (%)	27.6 (6.0)	28.7 (6.2)	0.5075	27.4 6.0)	28.3 (6.3)	0.6122	29.1 (6.9)	30.8 (5.1)	0.6899
RBC (×10^6^/μL)	4.3 (0.6)	4.6 (0.6)	**0.0290**	4.3 (0.5)	4.7 (0.6)	0.0592	4.1 (0.40)	4.5 (0.3)	0.8366
Hemoglobin (g/dL)	13.0 (1.9)	14.0 (1.8)	**0.0360**	13.2 (1.8)	14.3 (1.7)	**0.0393**	11.8 (1.8)	12.4 (1.0)	0.4845
Hematocrit (%)	39.5 (4.7)	42.3 (4.8)	**0.0268**	40.1 (4.6)	43.2 (4.7)	**0.0248**	36.9 (4.4)	38.2 (2.7)	0.6088
Platelets (×10^3^/μL)	216.0 (56.5)	211.7 (46.1)	0.7496	215.4 (61.9)	212.5 (46.4)	0.8554	218.9 (18.6)	207.4 (49.7)	0.6434
APTT (%)	97.1 (13.4)	96.3 (12.9)	0.8171	96.2 (12.1)	96.0 (13.7)	0.9551	101.4 (19.7)	97.9 (8.2)	0.7198
PT (%)	96.6 (13.2)	102.2 (14.5)	0.1611	86.1 (11.3)	102.7 (17.3)	0.1310	98.4 (21.8)	99.5 (12.8)	0.9303
PT/INR	1.04 (0.10)	1.01 (0.10)	0.2293	1.04 (0.09)	1.00 (0.11)	0.2641	1.04 (0.10)	1.01 (0.08)	0.6996
Fibrinogen (mg/dL)	327.8 (93.5)	319.6 (70.4)	0.7069	336.6 (98.2)	319.0 (74.1)	0.4818	285.4 (54.4)	322.8 (54.5)	0.3090
TP (g/dL)	7.3 (0.4)	7.33 (0.4)	0.6528	7.2 (0.4)	7.3 (0.5)	0.6805	7.5 (0.50)	7.6 (0.2)	0.8124
Albumin (g/dL)	4.3 (0.5)	4.33 (0.4)	0.9494	4.3 (0.60)	4.3 (0.4)	0.9010	4.6 (0.21)	4.4 (0.2)	0.3126
GOT (U/L)	20.8 (5.7)	23.3 (9.8)	0.2365	21.3 (5.9)	22.2 (8.0)	0.6390	18.4 (4.5)	28.6 (16.4)	0.2170
GPT (U/L)	20.9 (10.4)	26.2 (19.2)	0.1947	21.7 (10.3)	24.2 (11.6)	0.4303	16.8 (11.0)	36.0 (41.6)	0.3476
LDH (U/L)	198.1 (33.2)	185.8 (29.9)	0.1619	196.2 (34.9)	181.2 (24.6)	0.0975	212.3 (11.2)	213.5 (46.8)	0.9686
ALP (U/L)	233.6 (63.0)	241.3 (64.5)	0.6470	236.7 (57.9)	237.0 (62.2)	0.9828	219.0 (90.4)	262.4 (35.4)	0.4428
γ-GTP (U/L)	51.6 (63.2)	28.1 (10.2)	0.5916	60.5 (67.4)	29.0 (10.8)	0.1430	14.0 (0.1)	25.3 (0.1)	0.0771
Creatinine (mg/dL)	0.9 (0.2)	0.82 (0.2)	0.4937	0.9 (0.2)	0.9 (0.2)	0.4700	0.7 (0.2)	0.6 (0.0)	0.8480
Uric acid (mg/dL)	6.0 (1.7)	6.14 (1.6)	0.6891	6.1 (1.7)	6.5 (1.4)	0.4115	5.1 (1.3)	4.2 (1.2)	0.4009
BUN (mg/dL)	15.9 (4.9)	15.3 (3.3)	0.5500	15.9 (4.6)	15.5 (3.4)	0.7763	16.2 (6.6)	15.0 (2.6)	0.5092
Glucose (mg/dL)	122.5 (31.8)	129.2 (47.2)	0.5253	121.0 (33.4)	132.0 (49.6)	0.3690	130.0 (23.6)	115.4 (30.9)	0.4252
Calcium (mg/dL)	9.8 (0.3)	9.71 (0.4)	0.5741	9.8 (0.3)	9.7 (0.4)	0.3148	9.7 (0.3)	9.9 (0.1)	0.2483
e-GFR	71.4 (20.7)	74.9 (18.3)	0.4961	71.0 (20.8)	72.7 (14.0)	0.7287	73.4 (22.5)	85.2 (32.5)	0.5226
Urinary specific gravity	1.016 (0.006)	1.016 (0.006)	0.8782	1.016 (0.006)	1.016 (0.006)	0.9044	1.013 (0.006)	1.016 (0.009)	0.6434
Urinary pH	6.0 (0.7)	6.32 (0.8)	0.0899	5.9 (0.6)	6.3 (0.8)	0.0604	6.3 (1.2)	6.4 (0.8)	0.8783
Urinary RBC (/μL)	68.3 (37.8)	11.9 (5.5)	**0.0343**	80.1 (45.8)	5.7 (1.4)	**0.0108**	14.0 (9.1)	43.1 (30.9)	0.4647
Urinary WBC (/μL)	56.7 (38.7)	11.5 (7.4)	**0.0117**	54.5 (46.5)	3.6 (0.8)	**0.0036**	66.5 (40.5)	50.9 (43.4)	0.7540
Urinary epithelium (/μL)	4.0 (1.1)	2.66 (1.0)	0.1073	2.9 (1.0)	1.3 (0.2)	0.1428	9.4 (3.4)	9.7 (4.8)	0.6761
Urinary casts (/μL)	0.4 (0.1)	0.2 (0.1)	0.2339	0.5 (0.1)	0.1 (0.0)	0.1217	0.2 (0.2)	0.5 (0.4)	0.8345
Urinary bacteria (×10^3^/μL)	5.4 (3.0)	1.12 (0.2)	**0.0014**	2.4 (0.6)	0.9 (0.1)	**0.0010**	19.2 (16.8)	2.3 (0.9)	0.4647
Urinary volume (L/day)	1.4 (0.5)	1.6 (0.6)	0.1511	1.4 (0.5)	1.6 (0.3)	0.3038	0.9 (0.4)	1.5 (0.7)	0.2225

*SD* standard deviation, *SFs* stone formers, *NSFs* non-stone formers, *WBC* white blood cell, *RBC* red blood cell, *APTT activated partial thromboplastin time*, *PT* prothrombin time, *PT/INR* prothrombin time international normalized ratio, *TP* total protein, *GOT* glutamic oxaloacetic transaminase, *GPT* glutamic pyruvic transaminase, *LDH* l*actate dehydrogenase*, *ALP* alkaline phosphatase, *γ-GTP* γ-glutamyl transpeptidase, *BUN blood urea nitrogen*, *e-GFR* estimated glomerular filtration rate

The bold numbers indicate statistically significant differences (*P < 0.05) by Student’s *t*-test or the Mann–Whitney *U*-test

**Table 5:** Qualitative analysis of urinary protein and glucose

Parameter	Group	−, *n* (%)	±, *n* (%)	+, *n* (%)	++, *n* (%)	*P*-value*
Urinary protein	SFs	19 (63.3)	3 (19.0)	7 (23.3)	1 (3.33)	0.7007
	NSFs	20 (66.7)	5 (16.7)	4 (13.3)	1 (3.33)	
Urinary glucose	SFs	29 (96.7)	0 (0.0)	1 (3.3)	0 (0.0)	0.1785
	NSFs	23 (76.7)	1 (3.3)	3 (10.0)	3 (10.0)	

*SFs* stone formers, *NSFs* non-stone formers

*P < 0.05 indicates statistically significant differences by the Mann-Whitney *U*-test

### Aortic calcification rates

The aortic calcification rates of SFs and NSFs were 80.0 and 63.3%, respectively (Table 6). SFs tended to have an insignificantly higher incidence of aortic calcification (*P* = 0.3032); a similar tendency was observed in both sexes.

**Table 6:** Analysis of aortic calcification grades

	Group	Grade 0, *n* (%)	Grade 1, *n* (%)	Grade 2, *n* (%)	Grade 3, *n* (%)	*P*-value*
Total	SFs	6 (20.0)	13 (43.3)	6 (20.0)	5 (16.7)	0.3032
	NSFs	11 (36.7)	10 (33.3)	4 (13.3)	5 (16.7)	
Men	SFs	5 (20.0)	11 (44.0)	5 (20.0)	4 (16.0)	0.4672
	NSFs	8 (32.0)	10 (40.0)	2 (8.0)	5 (20.0)	
Women	SFs	1 (20.0)	2 (40.0)	1 (20.0)	1 (20.0)	0.3808
	NSFs	3 (60.0)	0 (0.0)	2 (40.0)	0 (0.0)	

*SFs* stone formers, *NSFs* non-stone formers

*P < 0.05 indicates a statistically significant difference by the Mann–Whitney *U*-test

### Morphology and composition of renal crystals

**Fig. 1:**
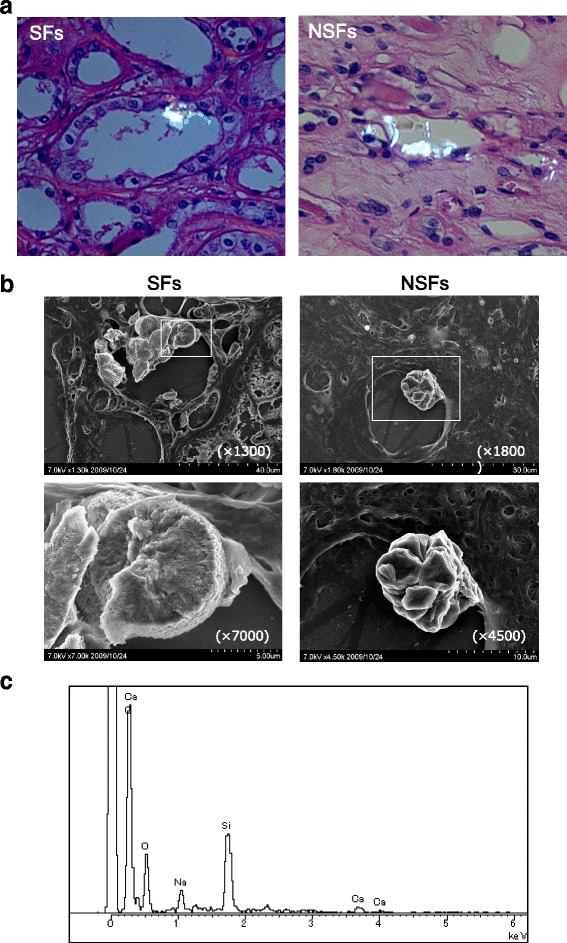
Morphology and composition of renal tubular crystal deposits in stone formers (SFs) and non-stone formers (NSFs). **a** Crystal attachment to the tubular walls detected by polarized light optical microphotography of hematoxylin and eosin-stained renal cortex sections (magnification, × 800). **b** Crystal attachment to the tubular walls detected by scanning electron microscopy (SEM) of the crystal ultrastructure. **c** Energy-dispersive X-ray spectroscopy (EDX) of the mineral components on the surface of SEM-detected crystal deposits. The EDX spectrum shows calcium as the main component of the deposits

Polarized light optical microphotography revealed renal crystal deposits with birefringence in both groups (Fig. 1a). SEM demonstrated no significant difference in crystal morphology and crystal attachment to the tubular walls between the groups (Fig. 1b). EDX showed that the main component of the deposits was calcium-containing crystals (Fig. 1c).

### Crystal transmigration

**Fig. 2:**
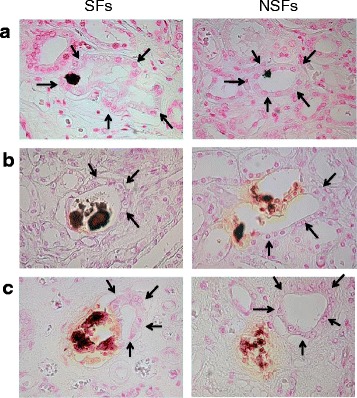
Microscopic observation of Pizzolato-stained calcium oxalate crystal deposits in the renal cortex, medulla, and papilla of stone formers (SFs) and non-stone formers (NSFs). **a** In the renal cortex, the crystals were located in the tubular lumen and attached to the walls. **b** In the medullary region, the crystal-attached tubular epithelial cells were abraded and crystal transmigration into the interstitium was observed. (**c**) In the papillary region, almost all the crystals were detected in the interstitium. Arrows indicate tubules with crystal deposits

Pizzolato staining revealed renal intratubular CaOx crystals (Fig. 2). The cortex crystals existed in the tubular lumen and adapted to the tubular walls (Fig. 2a). In the medullary regions, crystal-attached tubular epithelial cells were abraded and crystal transmigration into the interstitium was observed (Fig. 2b). In the papillary region, almost all crystals were detected in the interstitium (Fig. 2c). These findings were the same in both groups.

### Crystal distribution

**Fig. 3:**
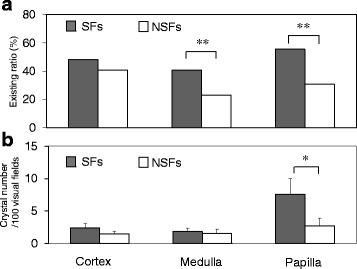
Comparison of the crystal distribution in the renal cortex, medulla, and papilla between stone formers (SFs) and non-stone formers (NSFs). **a** The existing ratios (number of kidneys with crystal formation/whole kidneys). **b** The crystal numbers per 100 visual fields (magnification, × 100). Data represent means (standard deviation); *P < 0.05 and **P < 0.01 indicates statistically significant differences by repeated-measures analysis of variance

In the renal cortex, the incidence ratios of SFs and NSFs were 48.1 and 40.7%, respectively (*P* = 0.3190; Fig. 3a). SFs had significantly higher incidence ratios in the medulla (40.9% vs. 23.1%, *P* = 0.0064) and papilla (55.6% vs. 30.8%, *P* = 0.0004). There were no significant differences in the number of crystal deposits (Fig. 3b) in the cortex (2.41 [0.63] vs. 1.44 [0.43], *P* = 0.3833) and medulla (1.84 [0.53] vs. 1.56 [0.63], *P* = 0.4079) between SFs and NSFs. However, SFs had a significantly higher number of papillary crystal deposits than NSFs (7.58 [2.42] vs. 2.75 [1.14], *P* = 0.0235). Furthermore, SFs had a significantly greater number of crystal deposits overall (*P* = 0.0187).

### Kidney stone-related gene and protein expression levels

**Fig. 4:**
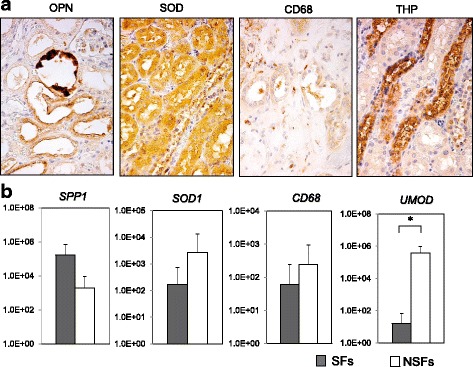
Kidney stone-related gene and protein expressions in stone formers (SFs) and non-stone formers (NSFs). **a** Immunohistochemistry (magnification, × 100) for osteopontin (OPN), superoxide dismutase (SOD), CD68, and Tamm–Horsfall protein (THP). **b** mRNA expression levels of the secreted phosphoprotein-1 gene (*SPP1*), *SOD1*, *CD68*, and uromodulin gene (*UMOD*) detected by quantitative polymerase chain reaction (qPCR). The glyceraldehyde 3-phosphate dehydrogenase gene was used as the internal control. Data represent means (standard deviation). *P < 0.05 indicates statistically significant differences by the Mann-Whitney *U*-test

OPN was expressed at the apical side of the distal tubular cells (Fig. 4a). SOD expression was diffusely detected among the proximal tubular cells. CD68-positive cells were detected mainly at the interstitial area of the renal papilla and tubular lumen. THP expression was diffusely detected among the distal tubular cells. As shown in Fig. 4b, SFs had a relatively high expression level of *SPP1* (*P* = 0.4959) and relatively low expression levels of *SOD1* (*P* = 0.0790) and *CD68* (*P* = 0.2764). Finally, SFs had a significantly lower expression level of *UMOD* (*P* = 0.0392) than NSFs.

### Multivariate analysis of the relationship between extracted factors and SFs

Multivariate logistic regression analysis was used to assess the relationship of the following factors with SFs: smoking habits, RBC, Ht, urinary RBC, urinary bacteria, existence of renal papillary crystals, and UMOD expression ratio. Continuous variables were adopted for analysis as two nominal scales with cut-off values set at the median values. The presence of renal papillary crystals was found to be a significant independent factor related to SFs (odds ratio 5.55, 95% confidence interval 1.08–37.18, *P* = 0.0395) (Table 7).

**Table 7:** Multivariate analysis for relationships between extracted factors and kidney stone formation

	OR (95% CI) For stone formers	*P*-value*
Smoking (+)	1.95 (0.40–9.92)	0.4037
RBC (≤ 4.6 × 10^6^/μL)	0.30 (0.03–2.38)	0.2518
Ht (≤ 48%)	1.28 (0.17–12.01)	0.8135
Urinary RBC (≤ 4.3/μL)	1.52 (0.28–8.29)	0.6211
Urinary bacteria (≤ 0.99 × 10^3^/μL)	4.46 (0.90–29.08)	0.0687
Renal papillary crystals (+)	5.55 (1.08–37.18)	0.0395
UMOD (existing ratio < 0.001)	4.15 (0.67–37.96)	0.1313

*P < 0.05 indicates a statistically significant difference. *OR* odds ratio, *CI* confidence interval, *RBC* red blood cell, *Ht* hematocrit, *UMOD* uromodulin

## Discussion

Kohri et al. [10] suggested that calcium kidney stone formation involves the expression of several stone matrix proteins, mainly OPN, in renal tubular cells, indicating that the phenomenon is inducible by both environmental and genetic factors [24]. However, their explanation has two major problems: (i) because kidney stone formation is asymptomatic, patients do not recognize its onset until colic pain occurs due to stone descent or chance detection by imaging studies; and (ii) due to the spread of extracorporeal shock wave lithotripsy, it has become difficult to extract tissues ethically from living kidneys, making it impossible to conduct detailed studies on kidney tissues, in contrast to the situation when open surgery was common. Due to the recent development of endoscopic instruments, morphological and pathological studies on Randall’s plaque have become more common. We recently conducted a genome-wide study of plaque tissue, resulting in the confirmation of inflammatory cytokine expression, increased immune cell number, and cellular apoptosis in renal papilla stone tissue [25]. However, these findings were limited to the renal papilla tissue and represent only the change in expression levels after stone formation. To resolve these problems, we enrolled patients with asymptomatic stones detected contingently by preoperative CT for the diagnosis of renal tumors and investigated renal parenchyma integrally using pathological whole-kidney samples. Specifically, we analyzed the crystal morphology and transmigration in addition to kidney stone-related gene and protein expression.

We found that SF group had a significantly higher smoking rate than NSFs. Słojewski et al. [26] did not detect significant correlations between smoking and kidney stone composition. Smoking is a significant, independent risk factor for atherosclerosis via the oxidative stress associated with mitochondrial damage [27]. Considering the similarity between kidney stone and atherosclerosis formation, [22] smoking might conceivably affect stone formation or crystal kinetics. The precise relationship between the risk of stone formation and smoking should be investigated in future studies.

SFs had significantly lower RBC, hemoglobin, and Ht values than NSFs. Renal ischemia via anemia could lead to renal tubular-cell injury, [28] implying that anemia might be involved in stone formation. However, patients with kidney stones have erythropoietin resistance caused by bone marrow oxalosis [29]. Furthermore, the increase in urine RBC, WBC, and bacterial counts may be the result of the erosion of the renal pelvic mucosa on Randall’s plaque in patients with stones [30]. Unfortunately, we did not consider the existence of plaque in this study.

The notable findings of this study are as follows: (i) regardless of kidney stone history, intratubular crystal deposits were detectable in the renal parenchymal tissues; (ii) the crystals transmigrated from the tubular lumen to the papillary interstitium; and (iii) SFs had a significantly higher number of crystal deposits in the renal papilla. Bergsland et al. [31] noted that SFs, especially those with idiopathic hypercalciuria, have higher urinary calcium molarity than NSFs and that the difference becomes significant at night. CaOx supersaturation but not calcium phosphate supersaturation is higher in SFs than in NSFs, which could also explain CaOx stone formation on papillary Randal’s plaques. CaOx crystal residues in the renal papilla could be another factor related to CaOx stone formation. Furthermore, Vervaet et al. [32] used hyperoxaluric rat model and human renal biopsy samples to indicate the gradual migration of intratubular crystals to the interstitium. In the hyperoxaluric mouse model we previously established, [11] intratubular crystal deposits were eliminated in about 6 days. The crystals were englobed and fragmented by macrophages and crystal deposits were undetectable in the renal papillary region at all time points. Boonla et al. [33] investigated MCP-1 and interleukin (IL)-6 messenger RNA expression in renal biopsy samples from SFs and extracted kidney samples from patients with renal cancer; they demonstrated relatively low MCP-1 and IL-6 expression levels in the cancerous samples compared to those in noncancerous tissues. In the present study, the significantly higher number of interstitial crystal deposits in the papilla of SFs and relatively high *CD68* expression level in NSFs suggest some important roles of macrophages in kidney stone prevention. The OPN, SOD, and CD68 expression levels were similar to those indicated in previous basic studies: SFs had increased OPN expression in the renal tubular cells, tubular-cell injury by oxidative stress, and reduced migration of renal macrophages. In particular, the significantly lower THP expression level in SFs indicates that THP has a crucial role as a kidney stone-preventive factor in humans.

On the basis of our results, we hypothesize the phenomena of human renal intratubular crystal processing. First, crystal nidi are generated in the tubular lumen of the renal cortex because of a urinary supersaturated condition [2, 3]. Some oxidative stresses, such as anemia or smoking, and renal tubular-cell injuries cause collapse of mitochondria and microvilli with decreased SOD expression [16–18]. Consecutively, OPN expression increases and THP downregulation induces crystal-cell interaction and the adaptation of aggregated crystals to the tubular epithelium [26]. Thereafter, the tubular epithelium disintegrates via apoptosis and crystal clusters transmigrate to the renal interstitium via the regenerating epithelium [32]. Tubular-cell injury increases the expression of MCP-1 or various chemokines, in turn inducing monocytes, their transmigration to the renal interstitium, and their differentiation into macrophages [20]. The interstitial crystals can then be removed by macrophages.

These calcification processes, including epithelial-cell injury via oxidative stress, the participation of OPN via inflammation, macrophage activity with phagocytosis, and processing and conversion of foam cells into calcified tissue, are similar to the processes of atherosclerosis formation [34]. SFs tended to have higher levels of aortic calcification. These outcomes suggest a new approach to kidney stone formation involving similar biomolecular processes to those involved in metabolic syndrome that are not related to kidney stone disease because of hyperuricemia, decreased urinary pH, or hypocitraturia caused by metabolic syndrome [35–37].

Multivariate analysis indicated that the presence of renal papillary crystals was significantly and independently related to stone formation. This result represents all of the relationships discussed above. These findings suggest the possibility that the process of kidney stone formation depends on some renoprotective abilities related to the processing of crystals formed in the renal parenchyma, especially the renal papilla.

This study has some limitations that should be discussed. We could not clarify how cancer background, involving environmental and genetic factors, affected “true” kidney stone formation. Furthermore, because this study was conducted retrospectively, detailed analysis of stone component and urinary biochemistry could not be performed. Moreover, Randall’s plaques were not detectable in the study sample.

## Conclusions

We identified similar phenomena to those detected in previous basic studies, such as crystal-cell interactions, increased OPN expression, decreased SOD and THP expression, and macrophage involvement in the human renal parenchyma. The new findings of this study were crystal formation in patients without kidney stones, crystal transmigration to the papillary interstitium, and crystal processing at the renal papilla regardless of stone formation. SFs may have reduced ability to eliminate renal parenchymal crystals than NSFs (especially in the papilla region), with associated gene expression changes.

## Abbreviations

CaOx: Calcium oxalate
CT: Computed tomography
EDX: Energy-dispersive X-ray spectroscopy
GAPDH: Glyceraldehyde 3-phosphate dehydrogenase
H&E: Hematoxylin and eosin
Ht: Hematocrit
IHC: Immunohistochemistry
IL-6: Interleukin-6
KSF: Kidney stone formation
MCP-1: Monocyte chemotactic protein-1
NSF: Non-stone former
OPN: Osteopontin
PBS: Phosphate-buffered saline
qRT-PCR: Quantitative reverse transcription-polymerase chain reaction
RBC: Red blood cell
RCC: Renal cell carcinoma
SEM: Scanning electron microscopy
SF: Stone former
SOD: Superoxide dismutase
SPP1: Secreted phosphoprotein-1
THP: Tamm–Horsfall protein
UMOD: Uromodulin
WBC: White blood cell
